# CORM-3 induces DNA damage through Ru(II) binding to DNA

**DOI:** 10.1042/BCJ20220254

**Published:** 2022-07-04

**Authors:** Rhiannon F. Lyon, Hannah M. Southam, Clare R. Trevitt, Chunyan Liao, Sherif F. El-Khamisy, Robert K. Poole, Mike P. Williamson

**Affiliations:** School of Biosciences, University of Sheffield, Firth Court, Western Bank, Sheffield S10 2TN, U.K.

**Keywords:** cancer, cisplatin, CORM, DNA damage, NMR, ruthenium

## Abstract

When the ‘CO-releasing molecule-3’, CORM-3 (Ru(CO)_3_Cl(glycinate)), is dissolved in water it forms a range of ruthenium complexes. These are taken up by cells and bind to intracellular ligands, notably thiols such as cysteine and glutathione, where the Ru(II) reaches high intracellular concentrations. Here, we show that the Ru(II) ion also binds to DNA, at exposed guanosine N7 positions. It therefore has a similar cellular target to the anticancer drug cisplatin, but not identical, because Ru(II) shows no evidence of forming intramolecular crossbridges in the DNA. The reaction is slow, and with excess Ru, intermolecular DNA crossbridges are formed. The addition of CORM-3 to human colorectal cancer cells leads to strand breaks in the DNA, as assessed by the alkaline comet assay. DNA damage is inhibited by growth media containing amino acids, which bind to extracellular Ru and prevent its entry into cells. We conclude that the cytotoxicity of Ru(II) is different from that of platinum, making it a promising development target for cancer therapeutics.

## Introduction

Organometallic complexes have long been of interest as medicinal drug targets [1]. Much of this interest centres around the platinum complex cisplatin, which (together with the related compounds carboplatin and oxaliplatin) is used in approximately half of cancer chemotherapies [1]. Cisplatin is cytotoxic because of its ability to bind covalently to DNA and crosslink it. There is however increasing incidence of resistance to cisplatin, spurring interest in other organometallics. One class of these is complexes of ruthenium.

Ruthenium exists in several oxidation states, of which the most interesting are Ru(II) and Ru(III): three Ru(III) complexes (NAMI-A, NKP-1339 and KP1019) have completed phase I clinical trials as anticancer drugs [2,3]. It is widely held that Ru(II) is the more cytotoxic oxidation state [4], and that the biological activity of Ru complexes depends heavily on the speciation of the complexes in solution: that is, on the chemical reactions undergone by the Ru ligands [2,3]. In solution, the most labile ligands tend to be halides, which dissociate rapidly to give aquated complexes [5]. Such complexes are generally found to be more soluble and more biologically active [6]. Here, we are specifically interested in Ru(II) carbonyl complexes, for which this is also true [7,8]. In blood, aquated Ru(II) complexes bind to albumin, and possibly also to the iron transport protein transferrin [9], although this has been linked more to Ru(III) than Ru(II) [2]. Binding to albumin is slow, taking many hours to reach equilibrium, and is reversible: binding to albumin has been shown to have very little effect on bioavailability, suggesting that the Ru is able to dissociate on reaching its biological target [10–13]. Albumin has therefore been described as a Ru depot [14]. Crystal structures of CORMs bound to proteins retain at least one carbonyl ligand [7,8,15], which may help in keeping the metal at a +2 oxidation level. It is still not clear how Ru complexes enter cells, though one possibility is that they use iron transport mechanisms [14]. Once inside cells, ruthenium is able to bind to proteins (via cysteine thiols [16,17] or histidines [18]), to free thiols such as glutathione [16,17], and also to DNA. In this behaviour it shows interesting differences as compared with platinum, which binds mainly to DNA, and tends to form square planar complexes, in particular linking the N7 positions of adjacent guanines, while ruthenium forms octahedral complexes [14]. The different reactivity of Ru as compared with Pt, and its greater range of biological targets, suggest that Ru may be able to evade tumour resistance better than Pt [14,19].

Ruthenium carbonyl complexes have been studied widely because of a completely different biological property: that they slowly release carbon monoxide, which itself has a wide range of biological properties. Here, we focus on one of these, carbon monoxide releasing molecule 3 [CORM-3], Ru(CO)_3_Cl(glycinate), which was designed as a rapid water-soluble carbon monoxide-releasing molecule and has been widely used in cellular studies as a source of CO [15,19,20]. For example, CORMs are being developed as drugs to deliver CO for specific therapeutic uses such as organ transplantation and treating pulmonary hypertension [21]. Numerous studies have shown that CORM-3 is toxic to bacterial cells but shows little toxicity to mammalian cells, which has stimulated research on modes of action of the Ru-based CORMs that have focussed on the toxicity of CO. However, recently we showed that CORM-3, as well as the related molecule CORM-2, in fact release very little CO in standard growth conditions [16,17,22]. Furthermore, we showed that the bacterial toxicity is due to the ruthenium(II) ion and probably not to CO at all. When CORM-3 or CORM-2 are dissolved in water, some of the Ru ligands (mainly the chloride ions as discussed above) are displaced by water or buffer, and some of the CO groups are attacked by hydroxide ions to give carboxylate ligands, producing a mixture of hydrated species [20,23]. These species are actively taken up by cells to reach high concentrations [3]. Once inside cells, they react with intracellular ligands. The most reactive ligands are thiols, such as glutathione and cysteine, which are present at high concentration inside most cells, and bind tightly (dissociation constant of the order of 1 µM) to ruthenium [16,17]. The effect of this intracellular sequestration of Ru complexes is to shift the equilibrium of hydrated Ru species from extracellular to intracellular, and produces high concentrations of Ru inside the cells, typically giving rise to intracellular Ru concentrations up to 30 to 100-fold greater than those in the medium [16,17]. One obvious consequence of the high intracellular concentration of Ru(II) is to sequester much of the free intracellular thiol, which one would expect to be a highly toxic effect, by affecting both the redox balance inside cells and glutathione-mediated detoxification mechanisms.

We have thus shown previously that binding of intracellular Ru to thiols is potentially toxic, easily sufficient to kill cells. We have however also previously shown that incubation of CORM-2 with *Escherichia coli* cells leads to binding of Ru to chromosomal DNA, at a level of ∼3 Ru per 1000 base pairs [17]. This would also be a potent mechanism for toxicity. We therefore here report studies using NMR on the binding of CORM-3-derived Ru(II) to DNA, and show that it binds to guanosine N7 positions, leading eventually to precipitation of the DNA. Using an alkaline comet assay, which measures single- and double-stranded DNA breaks, we show that CORM-3 causes a marked increase in DNA strand breaks in RKO cells. We therefore conclude that Ru (as generated by dissolution of CORM-3 in water) binds to DNA and leads to DNA strand breaks. The binding is different from that seen with cisplatin, which may allow Ru to be developed as an alternative anticancer treatment, for example in cases of cisplatin toxicity or resistance, a topic of considerable current interest [24].

## Results

### CORM-3 solutions bind to guanosine N7 in DNA

Binding of CORM-3-derived Ru(II) to DNA was assessed by NMR using a synthetic self-complementary B-DNA fragment, d(GTATGGCCATAC)_2_. This sequence was chosen because it contains a GpG sequence, which would allow 1,2-intrastrand binding, the most common binding site for the widely used anticancer drug cisplatin, Pt(NH_3_)_2_Cl_2_ [25]. It also contains a d(GpC) sequence, permitting 1,2-interstrand binding, also a common cisplatin binding mode.

**Figure 1.**
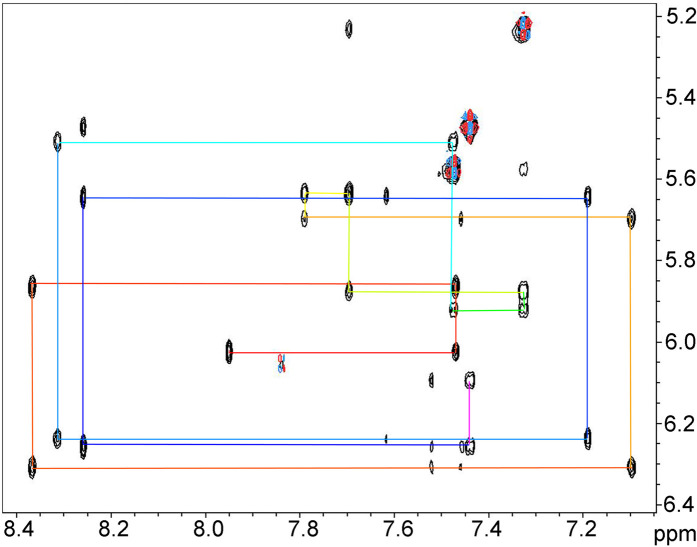
NOESY spectrum of the B-DNA 12-mer. An expansion of the region containing crosspeaks between H1′ (vertical axis) and H6/8 (horizontal axis) protons. 1.5 mM duplex, mixing time 60 ms. The lines show the sequential NOE walk in rainbow colours used to assign the spectrum, starting in red at the 5′ end.

**Figure 2.**
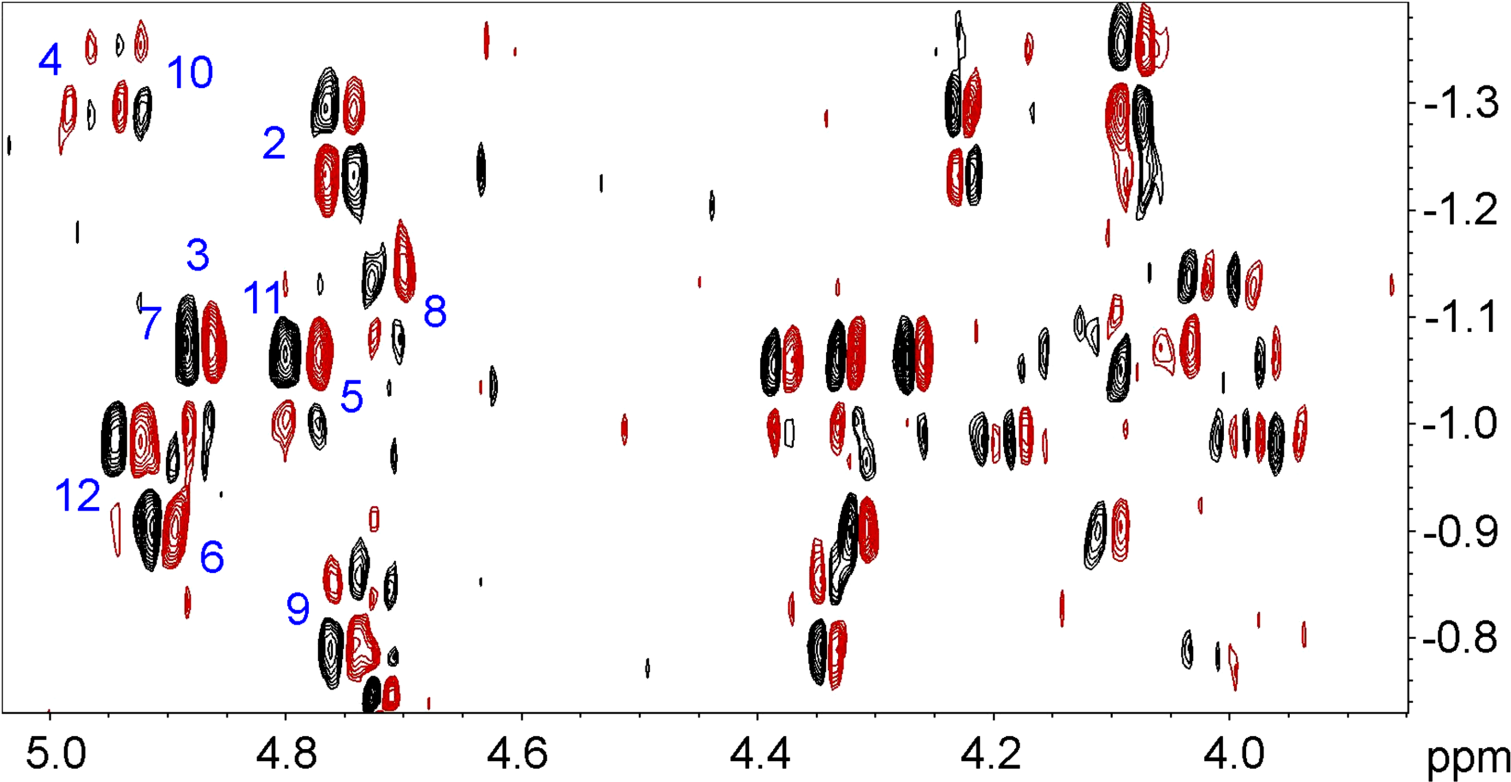
^31^P-^1^H COSY spectrum of the B-DNA 12-mer. Except for the phosphate group at the extreme 5′ end, each phosphate has crosspeaks to the 3′ proton at the upstream side, and the 5′ protons at the downstream side. Crosspeaks are numbered on the 3′ protons (4.5–5.0 ppm) and thus there is no crosspeak for residue 1.

**Figure 3.**
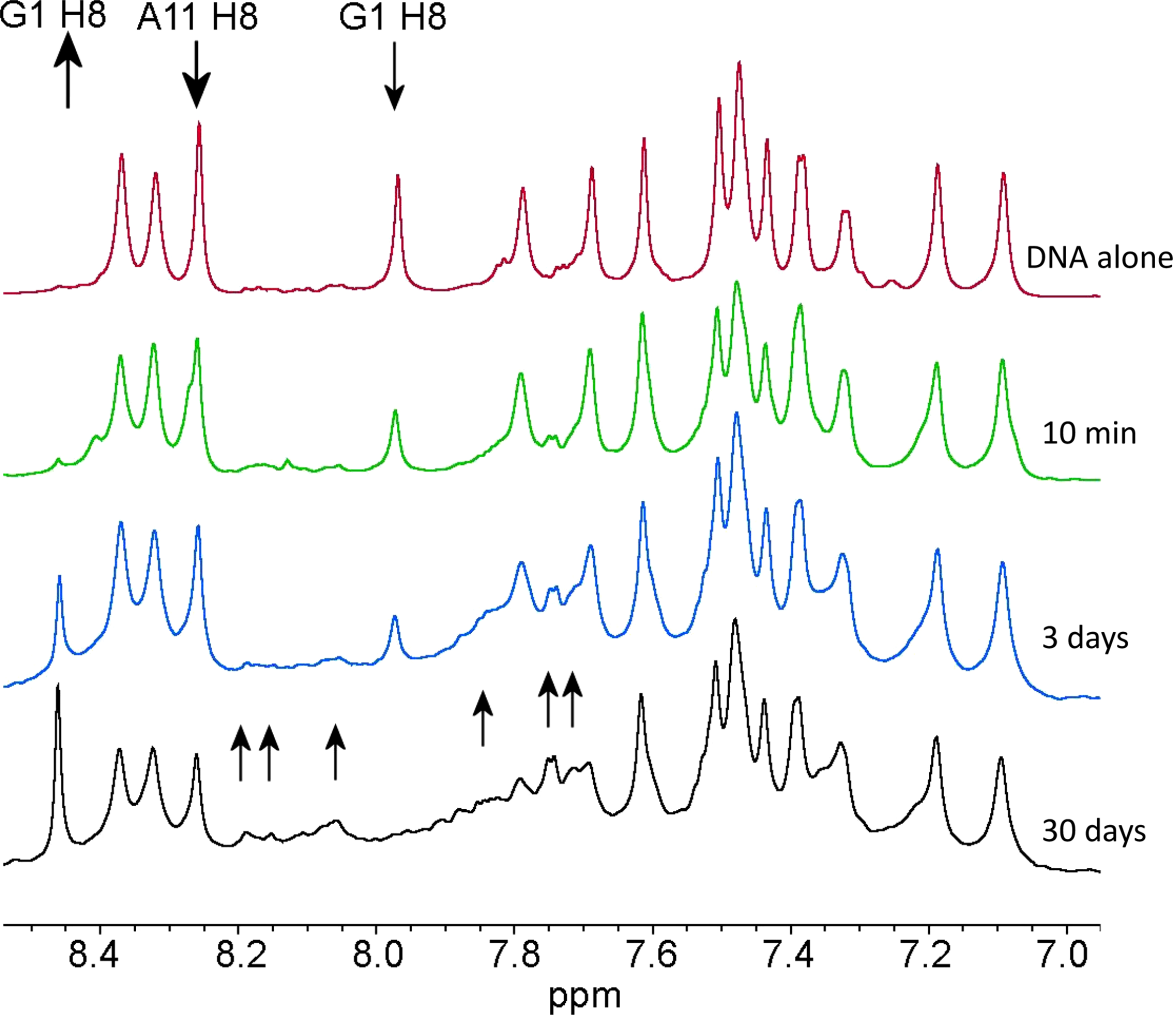
^1^H NMR spectra of the aromatic region of the oligonucleotide. Shown as a function of time after adding one equivalent of CORM-3. DNA duplex is 5 mM. Arrows indicate clear increases and decreases in intensity. The text at the right indicates the time since adding CORM-3.

**Figure 4.**
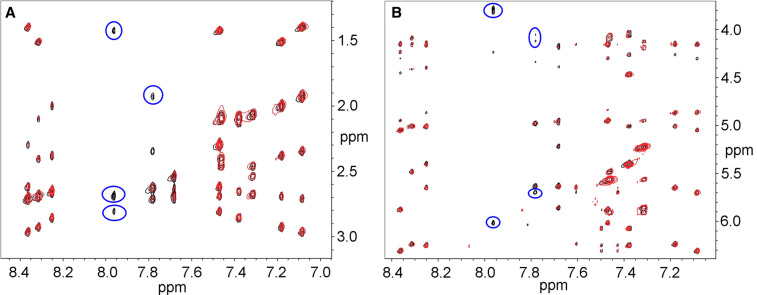
2D NOESY spectra of 5 mM 12-mer B-DNA duplex. Alone (black peaks) and after incubation for one month with CORM-3 (red). Peaks that have decreased significantly in intensity are circled. Expansion A shows crosspeaks between H6/8 and H2′/2″; expansion B shows crosspeaks between H6/8 and H1′/3′/4′/5′/5″.

The ^1^H NMR spectrum of the DNA sequence was assigned using standard homonuclear methods (Table 1 and Figure 1). The ^31^P spectrum was also assigned using ^31^P-^1^H COSY (Table 1, Figure 2). On addition of 1 equivalent of CORM-3, initial changes in the NMR spectrum were small, but after incubation for 3 days at 37°C, some large intensity changes were observable (Figure 3). The assignments of these signals were checked by 2D NMR (Figure 4). The sample was then left in the dark at 37°C for a further month, after which time, further changes could be seen in the NMR spectrum. These further changes were in most cases a completion of the changes seen at 3 days, although a number of lower intensity broad signals grew in intensity, indicating secondary reaction sites with some crosslinking of DNA molecules. Most of the spectral changes were monotonic with time, though a few appeared and then disappeared later, for example a small signal seen at 8.13 ppm at 3 h but not subsequently. There is thus evidence for equilibration of Ru between different sites on the DNA, with the resultant pattern being presumably the thermodynamic equilibrium.

**Table 1:** Chemical shift values of ^1^H and ^31^P in 12-mer B-DNA, 298 K, 5 mM in D_2_O

Residue	H1′	H2′	H2″	H3′	H4′	H2	H5	H6	H8	Me6	^31^P
G1	6.02	2.80	2.69	4.82	3.80				7.96		
T2	5.88	2.29	2.62	4.94	4.23			7.47		1.43	−1.27
A3	6.31	2.71	2.97	5.05	4.45	7.43			8.36		−1.03
T4	5.70	1.93	2.35	4.86				7.08		1.40	−1.33
G5	5.63	2.63	2.71	4.97	4.05				7.78		−1.02
G6	5.86	2.54	2.68	4.94	4.33				7.68		−0.93
C7	5.91	2.06	2.46	4.78			5.22	7.31			−1.03
C8	5.48	2.09	2.41	4.82			5.56	7.46			−1.11
A9	6.24	2.69	2.93	5.00		7.60			8.31		−0.83
T10	5.64	2.00	2.38	4.86	4.41			7.18		1.52	−1.33
A11	6.25	2.66	2.86	5.00	4.26	7.50			8.25		−1.03
C12	6.07	2.08	2.13	4.47	4.84		5.40	7.38			−0.95

**Figure 5.**
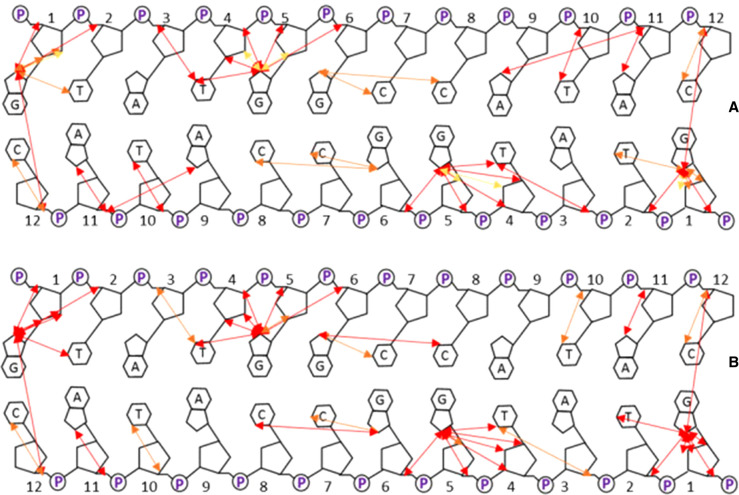
NOEs observed to decrease significantly in intensity on incubation of B-DNA 12-mer with one equivalent of CORM-3. (**A**) 3 days. (**B**) one month. Red arrows indicate NOEs that have completely disappeared; orange and yellow arrows are progressively smaller changes.

The NMR data are best summarised by mapping out the NOE connectivities that are affected significantly by incubation with CORM-3 (Figure 5). The overwhelming majority of NOEs missing or greatly reduced are those involving guanosine H8 protons, implicating binding of Ru(II) to the adjacent N7 positions, which is the expected binding site for transition metals to B-DNA [26,27], including that of Ru(II) complexes [28,29]. The signal most affected, and already dramatically weaker at 3 h, is the H8 signal from G1 (Figure 3), which is clearly the most reactive site. This is at the end of our synthetic nucleotide, and is thus considerably more sterically available than any of the others. It is worth noting that the signal for this proton in the Ru complex is the only new signal that we were able to assign unambiguously in the complex, at 8.46 ppm. It has few NOEs to sugar protons in the NOESY spectrum (Figure 4), implying that attachment of Ru leads to weakening of the interstrand hydrogen bonding pattern. The downfield shift of the G1 H8 signal is consistent with the binding of a strongly electron-withdrawing group such as Ru^2+^ at the adjacent N7 position [30].

The guanosine residue least affected by addition of one equivalent of Ru is G6 (Figure 5), which suffers only a small drop in intensity. This was a surprising observation, because with cisplatin this residue would be the most strongly affected, since it can make both 1,2 intrastrand and 1,2 interstrand crosslinks. This implies that Ru is not involved in intramolecular crosslinks to any significant extent, an observation previously made for monofunctional ruthenium(II) arene complexes [28,31]. In this behaviour, CORM-3 appears to behave differently from the related *cis*- and *trans-*[Ru(II)(DMSO)_4_Cl_2_], both of which have been reported to crosslink two adjacent N7 positions [32,33].

The ^31^P signal in DNA is sensitive to the double helix geometry, and changes markedly on either kinking or intercalation [34]. Neither was seen here, merely a loss in intensity of some ^31^P signals, mainly those adjacent to guanosines (data not shown). This confirms the ^1^H NMR result, implying structural weakening induced by Ru binding at isolated guanosine N7, but no crosslinking, kinking or intercalation of DNA.

**Figure 6.**
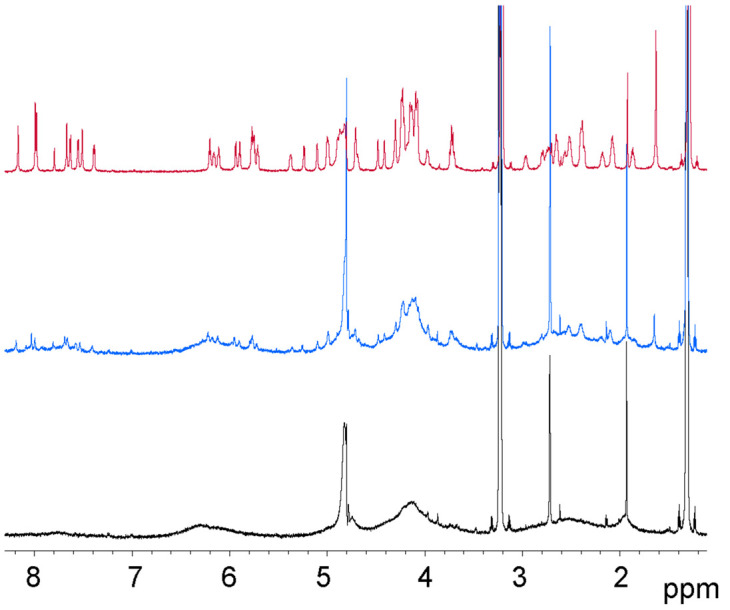
Time dependence of B-DNA NMR spectrum. Incubation of 1.2 mM B-DNA duplex with five equivalents of CORM-3 (10 equivalents of ruthenium). Top: DNA alone. Middle: immediately after addition of CORM-3. Bottom: one day later.

Addition of a large excess of CORM-3 led to the appearance of a large number of additional signals, but the most marked effect was a gradual reduction in signal intensity throughout the spectrum, and a marked broadening of peaks, implying crosslinking and aggregation of DNA (Figure 6). The NMR data therefore imply that Ru(II) binds at the most sterically accessible guanosine N7 sites, and does not make intramolecular crossbridges. At high concentration, it can gradually make intermolecular crossbridges, crosslinking different DNA double helices together, leading to broadening and loss of NMR signals.

### CORM-3 causes DNA damage in RKO cells

We have previously shown that incubation of *E. coli* cells with CORM-2 leads to binding of Ru to DNA, with a stoichiometry of ∼3 per 1000 base pairs, after 1 h incubation of cells with 30 µM CORM-2 [17]. This should be enough to cause significant DNA damage. We therefore carried out experiments to test whether incubation of CORM-3 with mammalian cells leads to observable DNA damage. We used an alkaline comet assay. In this experiment, cells are grown, trypsinised and then incubated in the desired conditions. They are subsequently embedded in agarose, lysed, and electrophoresed in an alkaline buffer, followed by staining and visualisation. The alkaline conditions denature the DNA [35] and allow both single and double-strand breaks to be seen, as faster moving comet tails in the stained gel. Initial experiments showed that concentrations of CORM-3 greater than ∼30 µM are too toxic to cells and gave unreliable results, so we used 20 µM CORM-3 and a short incubation time.

**Figure 7.**
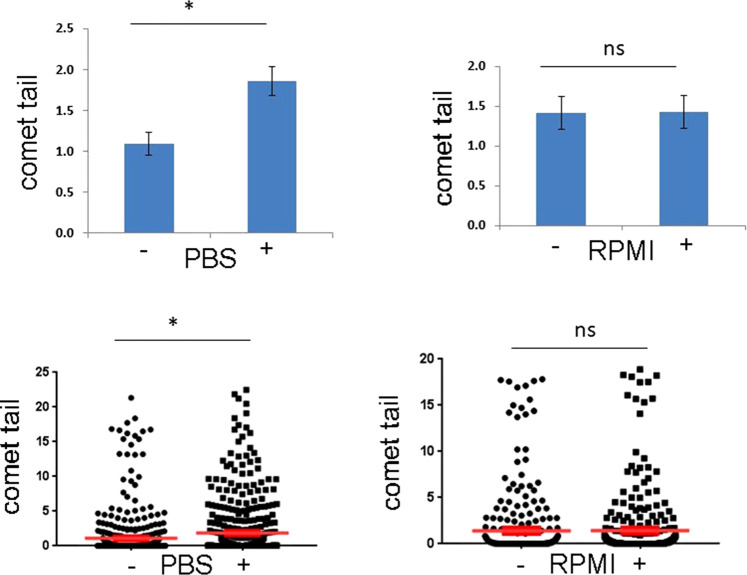
Comet assay for DNA damage. Top panels: mean tail moments for RKO cells treated with 20 µM CORM-3 for 30 min. Data show comet tails with and without CORM-3, incubated in PBS (left panel) and in RPMI (right panel). Error bars are SEM. Significance assessed by *t*-test, * *P* < 0.05, ns *P* > 0.05, *n* = 100. Bottom panels show range of tail moments. Moments greater than 22 were removed from the data because the cells are considered atypical.

Our previous results showed that incubation of mammalian cells in serum-containing media protected against DNA damage, because the amino acids in the serum bind to CORM-3 and prevent its accumulation into cells. We therefore grew RKO cells (derived from a human rectal carcinoma) in their preferred RPMI medium, trypsinised, and resuspended in medium. They were then diluted into either phosphate buffered saline (PBS) or into serum-containing medium and incubated with 20 µM CORM-3 for 30 min, after which they were embedded in an agarose gel, lysed, and electrophoresed in alkaline conditions. The results are shown in Figure 7, and show a significant increase in DNA damage in cells incubated in PBS, but no effect in cells incubated in serum-containing medium, which contains amino acids and thus prevents accumulation of CORM-3 into the cells. We conclude that CORM-3 leads to DNA damage over the relatively short timeframe of this experiment, which is less than 30 min.

## Discussion

We have previously shown that CORM-3 and CORM-2 kill cells not because of their released CO but because of the Ru(II) ion that they contain [16,17]. The ruthenium is concentrated inside cells, and binds to intracellular thiols, as found for example in glutathione and in cysteine residues on intracellular proteins. The results presented here confirm the cytotoxicity of Ru in CORM-3, and suggest that DNA damage is likely a major cause of the toxicity seen. In agreement with this finding, it has previously been shown that incubation of CORM-2 with *E. coli* leads to induction of the DNA repair gene *recA* [36], although Tavares et al. attributed this to an indirect response to the generation of reactive oxygen species (ROS). We note that the majority of studies on CORMs have seen a reduction in ROS, rather than an increase, following exposure to CORMs, but the data are unclear [3]. In light of our finding that very little CO is released from CORM-3 in typical growth conditions [16], a more likely explanation for the induction of *recA* is Ru-derived DNA damage.

CORM-3 and CORM-2 are useful sources of Ru(II) [3]. The ligands maintain ruthenium in a Ru^2+^ oxidation state. When dissolved in water, some of the ligands are replaced by water or by buffer, which makes the central Ru more accessible. This allows the CORMs to be taken up by cells, by a mechanism that is still not clear but may involve metal uptake systems. Ruthenium itself has no known natural biological function [3], so it has been suggested that it may be taken up using Fe^2+^ or Cu^2+^ uptake systems. However, it has also been suggested that the dipeptide permease/heme transporter DppABC may be involved [22]. Once inside the cell, the ruthenium reacts with intracellular nucleophiles, mainly thiols such as cysteine and glutathione. This alters the equilibrium of CORMs across the cell membrane and allows the ruthenium to attain much higher concentrations inside the cell than in the medium [16,17,37]. It would appear that the Ru(II) is sequestered inside the cell by thiol ligands, forming a reservoir of Ru(II), from which the Ru can be extracted under physiological conditions, for example to allow it to bind to DNA [38]. In [38] the bound thiol was readily oxidised, allowing it to be displaced by guanosine. However in general it is not clear to what extent thiol-bound intracellular Ru is available, for example to bind to DNA. The data presented here show that DNA binding is a slow and thermodynamically driven process, in comparison with thiol binding which is primarily kinetically driven. The implication is that the ruthenium transfers slowly (over a period of days) from thiols to DNA, although we have been unable to provide any clear proof that this occurs in our system.

The NMR results presented here demonstrate that the Ru(II) ion from CORM-3 binds to sterically accessible guanosine N7 positions, which appears to be generally the preferred binding site for Ru(II) [29,39]. The reaction is slow, taking several days to reach equilibrium at 37°C. We note that a similarly slow reactivity has been observed for other ruthenium complexes [40]. This is the same position that binds to cisplatin [26,27]. Our results however show a clear difference in that whereas cisplatin prefers intramolecularly bridged geometries, Ru(II) from CORM-3 clearly does not. The cytotoxicity of cisplatin is generally ascribed to its forming intramolecular crossbridges between pairs of purine N7, which leads to bending of DNA. Ruthenium does not do this, although it does form intermolecular crossbridges. Moreover, whereas platinum has affinity for adenosine [25], there is no indication from our results that ruthenium does so (see also [29]). The two metals therefore have a different molecular mechanism, which may provide a useful selectivity in both action and resistance [31]. In this context, it is worth noting that there have been several reports of Ru(II) complexes showing toxicity towards cisplatin-resistant cells [33,41–43], including for the related complex RuCl_2_(DMSO)_4_ [44]. They also have quite different kinetics, both for binding DNA and for entry to the cell. The aquated complexes formed from CORM-3 seem to behave differently from other aquated Ru(II) complexes [32,33]. It is thus clear that the specific ligand chemistry of a Ru(II) complex is critical for its biological effects.

Ruthenium compounds have been investigated as cancer therapeutics for decades, with general agreement that they show chronic rather than acute cytotoxicity, and that the nature of the complexation of the ruthenium ion outside the cell is critical for activity [45–48]. However, there has so far been little molecular understanding of the mode of action. We suggest that the insights from this study may be useful in refining research questions.

The slow binding equilibrium implies that on a cellular timescale, the cytotoxicity caused by CORM binding to DNA is unlikely to be reversible. It might therefore be expected to show extensive DNA damage, even if the amount of bound Ru is only of the order of one ion per 1000 base pairs or less. The results of the comet assay (Figure 7) show that extensive DNA damage does indeed occur, and (as expected) is retarded by incubation of cells in complex RPMI medium. The DNA damage seen here occurs within 30 min; thus, although the reaction with DNA is slow, it is very effective at causing DNA damage.

In this paper and recent work [16,17], we have shown that CORM-3 and CORM-2 have two significant biological effects: they sequester a high proportion of intracellular thiols, and they also induce damage in DNA. Both of these are likely to be harmful to cells. This may provide a novel mechanism for cell death in the context of cancer therapeutics, compared for example to cisplatin, which works entirely by DNA binding. The existence of a large body of work on effects of CORMs on cells (reviewed in [3,49]) provides a good basis to move forward rapidly.

## Experimental procedures

### Materials

The DNA oligonucleotide was synthesised by Eurofins Genomics (Germany). It was dissolved to 5 mM in 30 mM potassium phosphate pH 7.8, and annealed by heating to 85°C and slowly cooling. The sample was lyophilised and redissolved in 100% D_2_O. TSP (0.1 mM) was added as reference. CORM-3 was prepared as a stock solution of 5–20 mM in 30 mM deuterated potassium phosphate buffer pH 7.8. Solutions were stored at 4°C in the dark and used on the day of preparation.

### NMR experiments

NMR experiments were carried out on a Bruker Avance I (800 MHz, ^1^H) and Bruker Avance DRX (202 MHz, ^31^P) at 298 K. All homonuclear 2D experiments had 400 complex increments, with relaxation delays of 1.5 s. The mixing time for NOESY was 60–200 ms, and the spin lock time for TOCSY was 60 ms. The ^31^P COSY had 64 complex increments with a 1 s relaxation delay. Assignments were made using a combination of COSY, TOCSY and NOESY, with sequential NOE walks for 1′ and 2′/2″. Samples were left (at pH 7.8) at 37°C in the dark between NMR experiments and were monitored to check for lack of bacterial growth.

### Cell culture and comet assays

RKO cells were maintained as monolayers in RPMI medium supplemented with 10% foetal calf serum, 1% l-glutamine and 1% penicillin/streptomycin (Sigma) in a humidified atmosphere of 5% CO_2_ at 37°C. The comet assay followed Breslin et al. [50]. Confluent cells were washed with PBS, treated with 2 ml trypsin solution (37.5 ml PBS, 30 ml trypsin stock [1 mg/ml trypsin, 1 mM HCl, 20 mM CaCl_2_] and 7.5 ml 0.5 mM EDTA), and incubated for 2 min. Cells were centrifuged at 1000 rpm for 5 min, and resuspended in RPMI or PBS to a concentration of 3 × 10^5^ cells ml^−1^. An amount of 1 ml aliquots were taken, with half being treated with 20 µM CORM-3 and half being used as controls (30 min, 37°C). The cells were put on ice, centrifuged at 1000 rpm, and the pellet resuspended in 1 ml PBS. An amount of 150 µl of each sample was mixed with 150 µl 1.2% low-melting agarose at 42°C. An amount of 150 µl of this was pipetted onto a layer of set 0.6% normal agarose (150 µl) on a frosted glass cover slide, and a coverslip placed on top. The slides were left to set for 30 min at 4°C in the dark. The coverslips were then removed, and the slides were incubated in lysis buffer (2.5 M NaCl, 0.1 M EDTA, 10 mM Tris pH 10, 1% DMSO, 1% Triton X-100) at 4°C for 1 h in the dark. Slides were then placed in electrophoresis tanks in 1 mM EDTA, 50 mM NaOH, 1% DMSO and equilibrated for 45 m before running at 12 V for 25 m. Slides were neutralised by soaking overnight at 4°C in 0.4 M Tris pH 7.4, stained with SYBR green (Sigma) (1 : 20 000 dilution in PBS) for 5 m and scored using Comet Assay IV software. This measures the tail moment (tail length × % DNA in tail), a direct measure of the amount of DNA damage [51].

## Data Availability

All data described here are available on reasonable application to the corresponding author (M.P.W.).

## Abbreviations

CORM-3: carbon monoxide releasing molecule 3
PBS: phosphate buffered saline
ROS: reactive oxygen species
